# Ancient DNA Provides New Insights into the Evolutionary History of New Zealand's Extinct Giant Eagle

**DOI:** 10.1371/journal.pbio.0030009

**Published:** 2005-01-04

**Authors:** Michael Bunce, Marta Szulkin, Heather R. L Lerner, Ian Barnes, Beth Shapiro, Alan Cooper, Richard N Holdaway

**Affiliations:** **1**Henry Wellcome Ancient Biomolecules Centre, Department of ZoologyUniversity of OxfordUnited Kingdom; **2**Department of Anthropology, McMaster UniversityOntarioCanada; **3**Department of Ecology and Evolutionary Biology, University of MichiganAnn Arbor, MichiganUnited States of America; **4**Department of BiologyUniversity College LondonUnited Kingdom; **5**Palaecol ResearchChristchurchNew Zealand; Massey UniversityNew Zealand

## Abstract

Prior to human settlement 700 years ago New Zealand had no terrestrial mammals—apart from three species of bats—instead, approximately 250 avian species dominated the ecosystem. At the top of the food chain was the extinct Haast's eagle, *Harpagornis moorei. H. moorei* (10–15 kg; 2–3 m wingspan) was 30%–40% heavier than the largest extant eagle (the harpy eagle, Harpia harpyja), and hunted moa up to 15 times its weight. In a dramatic example of morphological plasticity and rapid size increase, we show that the H. moorei was very closely related to one of the world's smallest extant eagles, which is one-tenth its mass. This spectacular evolutionary change illustrates the potential speed of size alteration within lineages of vertebrates, especially in island ecosystems.

## Abstract 2

A phylogenetic analysis reveals the rapid increase in size of a New Zealand eagle, demonstrating the speed at which evolution can act on islands

## Introduction

**Figure 1:**
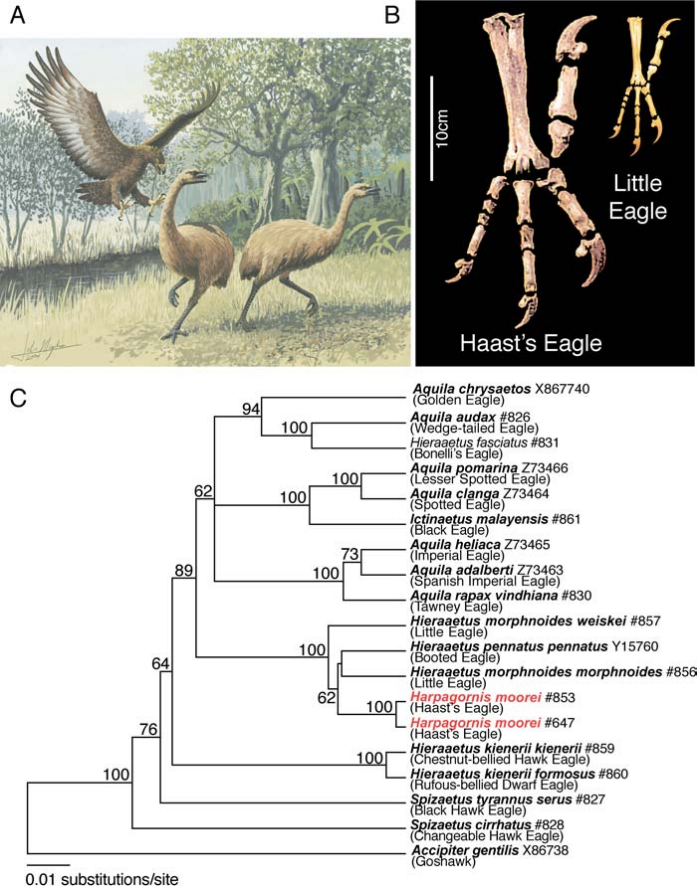
Images and Phylogenetic Analysis of New Zealand's Extinct Giant Eagle, H. moorei (A) An artist's impression of H. moorei attacking the extinct New Zealand moa. Evidence of eagle strikes are preserved on skeletons of moa weighing up to 200 kg. These skeletons show the eagle struck and gripped the moa's pelvic area, and then killed with a single strike by the other foot to the head or neck. (Artwork: John Megahan.) (B) Comparison of the huge claws of H. moorei with those of its close relative the Hieraaetus morphnoides, the “little” eagle. The massive claws of H. moorei could pierce and crush bone up to 6 mm thick under 50 mm of skin and flesh. (C) Maximum-likelihood tree based on cyt *b* data (circa 1 kb), depicting phylogenetic relationships within the “booted eagle” group. Extraction numbers or GenBank accession numbers are shown along with taxa name. Harpagornis moorei (red) groups exclusively with the small *Hieraaetus* eagles, and genetic distances suggest a recent common ancestor about 0.7–1.8 million years ago (early to mid Pleistocene). The tree uses an HKY + Γ_4_ + I likelihood model enforcing a molecular clock; maximum-likelihood bootstrap consensus values greater than 60% are shown.

Since the discovery of the first fossil in 1872 the sheer size of Harpagornis moorei (Figure 1A) has fuelled speculation about its evolutionary history, ecology, and extinction, which like many other New Zealand bird species, is linked ultimately to human arrival in the 13th century. Even though morphology indicates that H. moorei was approaching the upper limit of body mass for powered flight [1], it was still an efficient predator. Consistent with other members of the family Accipitridae, it killed by piercing and crushing its prey with its large talons (Figure 1B). Rock art, Maori oral history, and bone artefacts prove early Polynesians co-existed with the eagle; however, there is no evidence that humans were targets for this aerial predator. An exploratory skeletal analysis, using representative genera within the Accipitridae (but lacking Australasian representatives of the genera *Hieraaetus*), placed H. moorei as a sister species to *Aquila audax,* the Australian wedge-tailed eagle (circa 4.5 kg; 2 m wingspan) [2]. However, shifts in body size are common in island ecosystems [3] and may distort skeletal characters used in phylogenetic reconstructions.

## Results/Discussion

To further investigate the evolutionary history of this raptor we performed an ancient DNA study on the fossil remains of two extinct H. moorei specimens together with 16 extant eagles (Table S1). Short, overlapping segments of the mitochondrial cytochrome *b* (cyt *b*) and ND2 genes were PCR-amplified, sequenced and analysed with data available on GenBank to build a maximum-likelihood tree (Figures 1C and S1). Appropriate ancient DNA controls were undertaken [4], including multiple, overlapping amplifications and independent replication (see Materials and Methods). Surprisingly, the resulting phylogeny firmly placed the H. moorei in a “clade” with a group of small eagles of the genus *Hieraaetus, H. morphnoides,* the little eagle, and *H. pennatus,* the booted eagle (both circa 1kg; 1.2m wingspan), and not with A. audax. Moreover, the genetic distance separating H. moorei and the most recent common ancestor of the related *Hieraaetus* eagles is relatively small (1.25%). The lack of fossil calibration points for accipitrids precludes direct estimates of divergence times; however, when a molecular rate of 0.7%–1.7% per million years, as previously estimated for avian cyt *b* [5], is applied to the tree, a divergence estimate of approximately 0.7–1.8 million years ago is obtained. Although such indirect molecular dating estimates are error-prone, we believe that this range is the best available approximation of the “true” date when the lineages diverged (early to mid Pleistocene); however, additional molecular data and control region sequences may further clarify the topology and timing of the splits. The arrival of H. moorei into New Zealand's South Island appears to have been a recent event, probably involving a small bird-eating Asian/Australian *Hieraaetus* eagle that thereafter increased rapidly in size.

An analysis of mean body mass using independent contrasts clearly demonstrated that the size of H. moorei is an anomaly in the context of the eagle phylogeny shown in Figure 1C (see Materials and Methods). Factors that may have influenced such rapid morphological evolution include the size of potential prey, competition with smaller harriers (*Circus* spp.), and a complete lack of terrestrial predatory mammals in New Zealand. The speed and magnitude of the increase in body mass seems unique within vertebrate lineages, and is more significant because it occurred in a species still capable of flight. The avian faunas of Islands such as Hawaii (Hawaiian goose), the Galápagos (Galápagos finches), Mauritius (dodo), and New Zealand (moa) are often cited as examples of rapid evolution of flightlessness, shifts in body size, and other specialisations in birds [1,6,7]. Isolated island faunas derived from vagile colonists often have vacant niches for large herbivores and predators. In New Zealand the large herbivore niches were occupied for millions of years by the moa. Clearly, in the absence of mammalian predators, selection for large body size in an avian predator with a relatively generalised body form was not limited by competition. Other large predatory birds have evolved on islands in the absence of mammalian competitors, notably giant eagles and owls on Cuba [8], but the magnitude of the size increase in H. moorei over its sister taxa is unrivalled. H. moorei therefore represents an extreme example of how freedom from competition on island ecosystems can rapidly influence morphological adaptation and speciation.

The phylogeny in Figure 1C also reveals considerable problems with the current classification of the “booted eagle” group (eagles with feathered tarsi), especially the genera *Aquila, Hieraaetus,* and *Spizaetus,* which are clearly paraphyletic. Assignment of species within these genera has traditionally been problematic (see [9]), so this observation was not unexpected. However, it is apparent the name for the extinct New Zealand eagle should be amended to Hieraaetus moorei (Haast, 1872) (see Materials and Methods). The inclusion of H. moorei with the small *Hieraaetus* eagles implies the body size of the New Zealand species has changed by almost an order of magnitude since these lineages diverged. This spectacular evolutionary change illustrates the potential speed of size alteration within lineages of vertebrates and represents yet another example of the remarkable evolutionary processes that occur within island ecosystems.

## Materials and Methods

### 

#### DNA extraction and amplification

DNA was extracted and amplified from two H. moorei bones (Table S1) as previously described [10] using appropriate ancient DNA techniques. “Modern” toepad tissue (museum specimens) was extracted using Qiagen (Valencia, California, United States) DNeasy tissue extraction kits. Multiple negative extraction and amplification controls were included, to detect contamination. All PCR reactions were conducted as described in [10] using Platinum Taq HiFi (Invitrogen, Carlsbad, California, United States) together with the cyt *b* and ND2 primers listed in Table S2. Thermal cycling conditions were typically 40 cycles of 95 °C/55–60 °C/68 °C (30–45 s each). Sequences were determined using ABI Big Dye (v.3.1) on an ABI 3100 or 3730 (Applied Biosystems, Foster City, California, United States), according to manufacturer's instructions. Modern samples, and ancient samples subsequent to PCR amplification, were analysed in the Zoology Department, Oxford University. A single *Harpagornis* bone was sent to an ancient DNA facility at University College London (I. Barnes) for independent replication, where identical sequences were obtained for two cyt *b* amplifications. Similar cyt *b* and ND2 tree topologies, in addition to multiple overlapping sequences, make it unlikely that we are detecting a nuclear pseudogene.

#### Phylogenetic methods

Maximum-likelihood trees for cyt *b* and ND2 were selected using a heuristic search as implemented in PAUP*4.0b10 [11] under the HKY + Γ_4_ + I substitution model. The assumption of a molecular clock was tested using a likelihood ratio test in which the χ^2^ test statistic was two times the log likelihood difference between clock and non-clock models. For the cyt *b* tree the assumption of rate constancy was not rejected. Node support was evaluated for 1,000 bootstrap replicates. Bayesian Markov Chain Monte Carlo phylogenies were also generated on the cyt *b* dataset using BEAST [12] and MrBayes [13] under similar substitution models—the topology of these trees was consistent with Figure 1C and generated posterior support values higher than the bootstrap values.

Using the maximum-likelihood tree in Figure 1C, an independent-contrasts analysis was employed to determine whether correlations existed between phylogenetic position and body mass. Mean live weight estimates were obtained from the literature, and the average mass of H. moorei was estimated from femur length [2]. A test to measure the index of phylogenetic dependence was conducted; this measures the degree to which traits vary across taxa (in a phylogeny) in accordance with predictions of a neutral Brownian model according to [14]. The results (not shown) clearly demonstrate that the mass of H. moorei is clearly an “outlier” in the context of the phylogeny presented here.

#### *Hieraaetus* systematics

The type species for the genus *Hieraaetus* is H. pennatus (Gmelin, 1788); therefore, the taxa grouping strongly with H. pennatus must remain in that genus. The close genetic relationship of H. morphnoides with H. pennatus firmly embeds this species in *Hieraaetus*. However, the New Guinea subspecies presently recognised as *H. morphnoides weiskei* is genetically, geographically, and morphologically distinct and warrants species status, which necessitates the new combination Hieraaetus weiskei (Reichenow, 1900). Harpagornis moorei is included in the clade with H. pennatus and *H. morphnoides,* and hence its generic assignment must reflect that. The name for the extinct Harpagornis moorei of New Zealand should therefore be amended to Hieraaetus moorei (Haast, 1872).

## Supporting Information

Figure S1 Maximum-likelihood tree generated using 434 bp of ND2 data from a subset of eagle taxa. The tree topology seen here is identical to that seen in Figure 1C and is an independent verification of the tree topology (89 KB PDF).

Table S1 List of Eagle Taxa Used in This Study along with Museum Accession Numbers and Sample Provenance (64 KB PDF).

Table S2 List of Avian cyt *b* and ND2 Primers Used in This Study (54 KB PDF).

### Accession Numbers

Sequences have been deposited in GenBank (http://www.ncbi.nlm.nih.gov/Genbank/index.html) under accession numbers AY754044 to AY754056.

## Abbreviations

cyt *b*: cytochrome *b*
