# Supplementary material for: Ancient DNA Provides New Insights into the Evolutionary History of New Zealand's Extinct Giant Eagle
Source: PLoS Biol. 2005 Jan 4;3(1):e9. doi: 10.1371/journal.pbio.0030009 (PMC539324; doi:10.1371/journal.pbio.0030009)
Supplement: Figure S1 — (89 KB PDF). [file pbio.0030009.sg001.pdf]

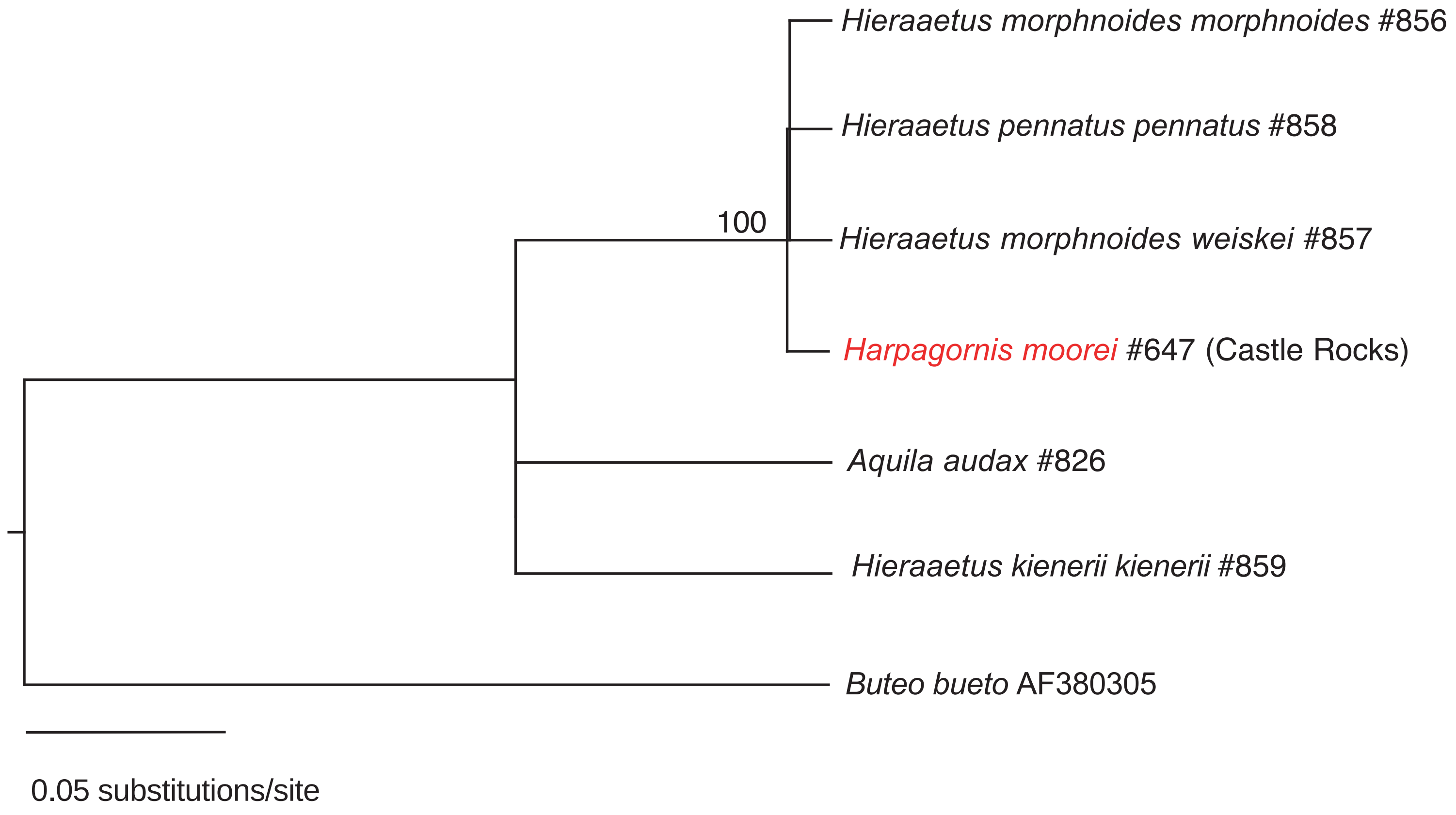

Figure S1. Maximum likelihood tree generated using 434bp of ND2 data from a subset of eagle taxa. The tree topology seen here is identical to that seen in Figure 1C and is an independent verification of the branching order.
