# Supplementary material for: Ancient DNA Provides New Insights into the Evolutionary History of New Zealand's Extinct Giant Eagle
Source: PLoS Biol. 2005 Jan 4;3(1):e9. doi: 10.1371/journal.pbio.0030009 (PMC539324; doi:10.1371/journal.pbio.0030009)
Supplement: Table S1 — (64 KB PDF). [file pbio.0030009.st001.pdf]

Species	Sample No.	Country of Origin	Collection date	Museum details	Tissue
Harpagornis moorei (Sample replicated in London)	#853	Mt Owen, Nelson, New Zealand	2096 years BP	Museum of New Zealand S27773	Bone
Harpagornis moorei	#647	Castle Rocks,Otago, New Zealand	Unknown (Estimated between 300-3000 years)	Museum of New Zealand DM2134	Bone
Aquila audax	#826	Australia	1879	(OUMNH) B19505	Toepad
Spizaetus tyrannus serus	#827	Equador	1936	OUMNH B1721	Toepad
Spizaetus cirrhatus limnaetus	#828	Borneo	1878	OUMNH B9493	Toepad
Aquila rapax vindhiana	#830	India	1963	OUMNH B5536	Toepad
Hieraaetus fasciatus fasciatus	#831	No provenance Distribution: S. Europe, N. Africa, Asia	1957	OUMNH B3762	Toepad
Hieraaetus morphnoides morphnoides	#856	Australia	1968	NHM, Tring 1969.4.22	Toepad
Hieraaetus morphnoides weiskei	#857	New Guinea	1908	NHM, Tring 1913.3.6.35	Toepad
Hieraaetus pennatus pennatus	#858	India	1873	NHM, Tring 85.8.19.1302	Toepad
Hieraaetus kienerii kienerii	#859	No provenance Distribution: Asia	1877	NHM, Tring 85.8.19.1331	Toepad
Hieraaetus kienerii formosus	#860	Thailand	1914	NHM, Tring 1914.9.22.2	Toepad
Ictinaetus malayensis malayensis	#861	No provenance Distribution: Southeast Asia	1932	NHM, Tring 1932.12.21.35	Toepad

Table S1: Eagle samples used in this study. These taxa, in addition to Genbank accession numbers X867740, Z73466, Z73464, Z73465, Z73463, Y15760, X86738 and X86741 were used in this study.
