# Supplementary material for: Ancient DNA Provides New Insights into the Evolutionary History of New Zealand's Extinct Giant Eagle
Source: PLoS Biol. 2005 Jan 4;3(1):e9. doi: 10.1371/journal.pbio.0030009 (PMC539324; doi:10.1371/journal.pbio.0030009)
Supplement: Table S2 — (54 KB PDF). [file pbio.0030009.st002.pdf]

Primer name	Primer Sequence (5' to 3')	MtDNA gene
eagle-cytb-5f	CCYTNCTAGGAATCTGCCTA	Cytochrome *b*
eagle-cytb-271r	CCTACGAAGGCRGTTGCTAT	Cytochrome *b*
eagle-cytb-243f	CACAGGRATCATTCTCCTACT	Cytochrome *b*
eagle-cytb-566r	ATGTCYTTTARGGAGAAGTATG	Cytochrome *b*
eagle-cytb-543f	CTGTGACAAAATCCCATTCCA	Cytochrome *b*
eagle-cytb-795r	TACTGAGGCAGCTAGRGCTAG	Cytochrome *b*
eagle-cytb-1f	TACTTCCTATTYGCATACGC	Cytochrome *b*
eagle-cytb-3r	TGRRGAAGTAGGTGAGGGA	Cytochrome *b*
eagle-cytb-890f	CCAACCTCCTCATCCTCACAT	Cytochrome *b*
eagle-cytb-thr-1069r	CTTCAGTTTTTGGTTTACAAGACC	Cytb/tRNAthr
L5219 trna-met-40	CCCATACCCCGAAAATGATG	ND2
Eagle-nd2-247r	TGGGTTARTTGGGTRATGTCYCATTG	ND2
Eagle-nd2-852f	CTCTCCCTNCTAGGNTTATTYTTCTACC	ND2
H6313 trna-trp+40	CTCTTATTTAAGGCTTTGAAGGC	ND2

Table S2. Primers used in this study. Primers were designed from mitochondrial sequences deposited on Genbank and from previous studies (Sorenson 1999).
